# The role and medical prospects of long non-coding RNAs in cardiovascular disease

**DOI:** 10.1007/s10741-023-10342-1

**Published:** 2023-10-05

**Authors:** Najung Kim, Woo-Young Chung, Je-Yoel Cho

**Affiliations:** 1 Department of Biochemistry, BK21 Plus and Research Institute for Veterinary Science, College of Veterinary Medicine, Seoul National University, 08826 Seoul, Republic of Korea; 2 Comparative Medicine Disease Research Center, Seoul National University, 08826 Seoul, Republic of Korea; 3 Department of Internal Medicine, Boramae Medical Center , Seoul National University College of Medicine, Seoul National University, Boramaero 5 Gil 20, Dongjak-Gu, Seoul, Korea

**Keywords:** lncRNA, Cardiovascular disease, RNA-based therapy, Therapeutic targets

## Abstract

Cardiovascular disease (CVD) has reached epidemic proportions and is a leading cause of death worldwide. One of the long-standing goals of scientists is to repair heart tissue damaged by various forms of CVD such as cardiac hypertrophy, dilated cardiomyopathy, myocardial infarction, heart fibrosis, and genetic and developmental heart defects such as heart valve deformities. Damaged or defective heart tissue has limited regenerative capacity and results in a loss of functioning myocardium. Advances in transcriptomic profiling technology have revealed that long noncoding RNA (lncRNA) is transcribed from what was once considered “junk DNA.” It has since been discovered that lncRNAs play a critical role in the pathogenesis of various CVDs and in myocardial regeneration. This review will explore how lncRNAs impact various forms of CVD as well as those involved in cardiomyocyte regeneration. Further, we discuss the potential of lncRNAs as a therapeutic modality for treating CVD.

## Introduction

The Human Genome Project started in 2001 [1] and was 99% completed in 2004 [2], and during this time it became clear that only approximately 1.5% of the human genome coded for approximately 21,000 genes [3]. Non-coding DNA, which does not code for a protein, was termed “junk DNA” [4]; however, with the development of genome tiling array technology, The Encyclopedia of DNA Elements (ENCODE) project in 2012 found that RNA transcripts arose from at least 76% of the human genome and these RNA molecules likely had biological functions [5]. According to the Human GENCODE project, there are more than 16,000 currently identified long noncoding RNAs (lncRNAs) [6] defined as RNAs that do not code for proteins and are longer than 200 nucleotides in length [7]. lncRNAs are primarily transcribed by RNA polymerase II and are classified into five types according to their structural location: sense, antisense, bidirectional, intronic, and intergenic lncRNAs [5].

lncRNAs perform multiple functions through various mechanisms. At the transcriptional level, lncRNAs function as signals, decoys, guides, and scaffolds. By influencing signaling factors or acting by themselves, lncRNAs can regulate downstream genes. lncRNAs can also negatively regulate downstream genes by decoying protein molecules to block specific molecular pathways, acting as guides for the localization of specific molecules to particular locations, and/or functioning as scaffolding that favors the association of various proteins to promote function as a macromolecular complex [5, 8]. At the post-transcriptional level, lncRNAs can regulate mRNA splicing by binding to proteins that modulate mRNA turnover, and/or stabilize and translate mRNA transcripts by binding to target RNA. lncRNAs also play a role in sponging miRNA, with sequestration of microRNAs, and serve as competing endogenous RNA (ceRNA) to regulate mRNA expression [7, 9]. Transcriptional genome-wide studies (TWAS) have found that lncRNAs contribute to both human disease and genetic traits. TWAS and co-localization analysis identified 14,100 lncRNAs from 49 tissues contributing to 101 genetic traits [10]. It has been reported that lncRNAs regulate gene action during cardiovascular disease (CVD) and cardiac development. Examining how lncRNAs are produced and regulated in disease states is crucial to finding clues for novel approaches to disease treatment.

CVD is a condition in which either heart or blood vessels are structurally or functionally abnormal. Although numerous pharmacological and interventional therapies for CVD have been developed to date, and the World Health Organization (WHO) has instituted the “Global Action Plan for the Prevention and Control of Non-communicable Diseases (NCDs) 2013–2020” mission, CVD remains a leading cause of morbidity and mortality worldwide [11]. According to National Health and Nutrition Examination Survey (NHANES) data, the prevalence of CVD in adults aged 20 years or older was 49.2% [12]. WHO estimates that 17.9 million people died from CVD, which represents 32% of all deaths worldwide, and 37% of non-communicable diseases among those under 70 years of age in 2019 [13]. The Global Burden Disease (GBD) 2020 study shows an estimated 19.05 million people died in 2020 from CVD, an 18.71% increase from 2010 [14].

By analyzing lncRNAs and understanding their molecular basis for various forms of CVDs such as MI, cardiac fibrosis, and cardiomyopathy (Table 1), as well as analyzing their role in cardiomyocyte regeneration through controlling proliferation and development (Table 2), and by exploring how lncRNAs can be effectively harnessed for the treatment of CVD, we may be able to identify new therapeutic targets for treating this common form of disease.

**Table 1:** LncRNA that plays a role in CVD

Disease	Mechanism	LncRNA	Target gene	Reference
Myocardial Infarction	Guide	Caif	Myocardin	[101]
		Cpr	Mcm3	[102]
	Signal	Mirt1	P65	[103]
		*Snhg1*	PTEN	[20]
	CeRNA	Carl	miR-539	[21, 22]
		Oip5-As1	miR-29a	[24, 104]
		Chrf	miR-182-5p	[23]
		Hotair	miR-206	[26]
		Apf	miR-188-3p	[105, 106]
		Carel	miR-296	[107]
		Mirf	miR-26a	[108]
		Ftx	miR-29b-1-5p	[109]
		AZIN2-sv	miR-RNA-214	[110]
		Gpr19	miR-324-5p	[111]
		CRRL	miR-199a-3p	[54]
		Uca1	miR-143	[112]
Heart Fibrosis	ceRNA	Malat1	miR-145	[32]
		Miat	miR-24	[33]
	Decoy	Meg3	Mmp2	[30]
	Guide	Wisper	Plod2	[31]
Cardiomyopathy	Scaffold	Mhrt	Brg1	[36–38]
		Ahit	Mef2a	[113]
		Chaer	hypertropic genes	[40]
	Signal	Chast	Plekhm1	[41]
	Guide	H19	Tesc	[114]
		ZNF593-AS	RYR2	[45]
	ceRNA	Chrf	miR-489	[115]
		Neat1	miR-19a13p	[42]
		Plscr4	miR-214	[116]
		Hotair	miR-19	[117]
		Miat	miR-93, miR-150	[118, 119]
		Magi1-IT1	mir-302e	[120]
		Syne1-As1	miR-525-5p	[121]
		Xist	miR101, miR330-3p	[122]
		H19	miR-675	[46]

**Table 2:** LncRNA that plays a role in cardiomyocyte regeneration

LncRNA that activates cardiac proliferation			
Mechanism	LncRNA	Target gene	Reference
Signal	ECRAR	E2F1	[50]
ceRNA	NR_045363	miR-216a	[52]
mRNA stabilization	Sirt1-as	Sirt1	[51]
LncRNA that suppress cardiac proliferation			
Mechanism	LncRNA	Target gene	Reference
Scaffold	Cpr	MCM3	[102]
	Sarrah	NRF	[123]
Signal	LncDACH1	YAP1	[53]
ceRNA	CAREL	miR-296	[107]
	CRRL	miR-119a-3p	[54]
	AZIN2-sv	miR-214	[102]
LncRNA in cardiomyocyte differentiation			
Mechanism	LncRNA	Target gene	Reference
Guide	Bvht	MesP1	[55]
Scaffold	Linc1405	MesP1	[56]
	Upperhand (uph)	Hand2	[124]

## lncRNAs in failing heart and cardiac regeneration

### lncRNAs in myocardial infarction

**Fig. 1:**
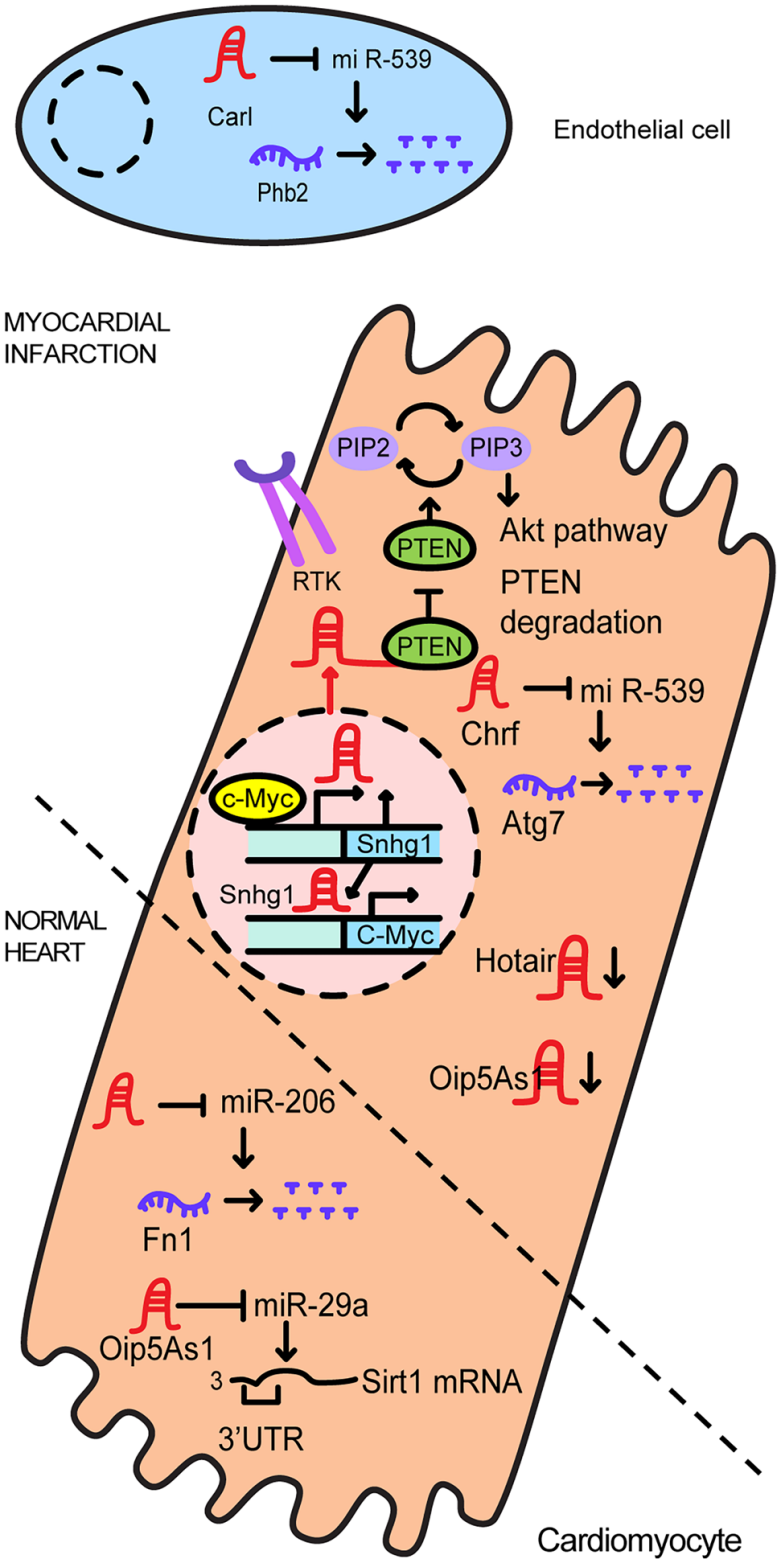
The regulatory functions involved in myocardial infarction

Myocardial infarction (MI) occurs when the myocardium is deprived of oxygen for a prolonged period of time, resulting in hypoperfusion and necrosis, which can result in sudden death. Due to its sudden onset, MI is also referred to as “acute” MI [15]. According to surveys conducted by the Atherosclerosis Risk in Communities (ARIC) study between 2005 and 2014, there are 605,000 new cases of MI each year and 200,000 cases of recurrent MI. Furthermore, the MI incidence rate is higher in men than in women with an age of first onset of 65.5 years in men and 72.0 years in women [16]. Certain data indicate a higher occurrence of MI in men, attributed to differences in lifestyle and customs. Men often have more demanding occupations and employ distinct stress management techniques compared to women. They may also pay less attention to maintaining a healthy diet, have a higher likelihood of being overweight, and might be less mindful of their own symptoms and well-being [17]. Consequently, when compared to women with similar risk factors, men with hypertension, high BMI (body mass index), and type II diabetes experience higher rates of MI [18]. Although MI is more common in men than in women throughout their lives, the gender differences in MI risk tend to reduce as individuals age [19]. Figure 1 illustrates the mechanisms through which lncRNAs associated with myocardial infarction function.

In the nucleus of cardiomyocytes affected by MI, the expression of lncRNA small nucleolar RNA host gene 1 (*Snhg1*) increases within in the damaged heart tissue*. Snhg1* acts as a signal molecule by decoying phosphatase and tensin homolog (PTEN) to regulate signal pathways. After MI, *Snhg1* is mainly expressed in cardiomyocytes and binds to the PTEN protein, resulting in its degradation and consequential activation of PI3K/Akt signaling. c-Myc, a downstream protein in the PI3K/Akt signaling pathway, forms a positive feedback loop with Snhg1 by binding to the Snhg1 promoter. Consequently, Snhg1 regulates cardiomyocyte proliferation after MI by upregulating PI3K/Akt signaling and promoting an angiogenic response through the induction of VEGFA gene expression [20].

Within the cytoplasm, lncRNA can sponge miRNA molecules and thus play an important role in angiogenesis, mitochondrial dynamics, apoptosis, and cell viability by sequestering molecules. The lncRNA AK017121 (UCSC Genome Browser on Mouse, 2011 Assembly) is also termed the cardiac apoptosis-related lncRNA (*Carl*) and is highly expressed in cardiomyocytes injured following MI ischemia. *Carl* sponges miR-539 by directly binding to it and this reduces infarct size during MI as miR-539 suppresses *prohinin2 (Phb2)* expression by binding to *Phb2* mRNA and the Phb2 protein functions in inhibiting mitochondrial fission and apoptosis. To put it concisely, *Carl* regulates mitochondrial dynamics through the *Carl*/miR-539/*Phb2* signaling axis [21, 22].

Another example of an important lncRNA in the heart is cardiac hypertrophy-related factor (*Chrf*). This lncRNA is expressed not only in cardiac hypertrophy but also in myocardial I/R injury models. In the context of myocardial I/R injury, silencing of *Chrf* reduces autophagy, inhibits apoptosis, increases cell viability, and reduces lactate dehydrogenase (LDH) levels. *Chrf* appears to limit myocardial I/R injury by preventing Atg7 from being inhibited by miR-182-5p [23].

The lncRNA *Opa-interacting protein 5-antisense transcript 1 (Oip5-as1)* is also downregulated in cardiomyocytes in response to oxygen–glucose deprivation/reoxygenation (OGD/R), an in vitro MI injury model. *Oip5-as1* plays a role in reducing oxidative stress in OGD/R conditions by acting as a ceRNA and sponging miR-29a and subsequently preventing miR-29a from binding to the 3′ UTR of the sirtuin-1 (Sirt1) mRNA. This action results in the activation of the Sirt1/5′ AMP-activated protein kinase (Ampk)/peroxisome proliferator-activated receptor gamma coactivator 1-alpha (Pgc1-α) pathway. In this pathway, Sirt1 is known to suppress oxidative stress and apoptosis during MI; Ampk is involved in energy metabolism which aids cell survival following ischemic injury; and Pgc1-α is responsible for energy homeostasis, oxidative metabolism, and cardiac mitochondrial function. Such functions of the Sirt1/Ampk/Pgc1-α pathway benefit survival of cardiomyocyte cells during MI injury [24].

HOX antisense intergenic RNA (*Hotair*) is decreased during acute myocardial infarction (AMI) both in a mouse model and human patient serum. *Hotair* prevents hypoxia-induced cardiomyocyte apoptosis [25], and since *Hotair* has a binding site for miR-206, it prevents inactivation of *fibronectin 1* (*Fn1*) by sponging miR-206. The role of *Fn1* in cardiac vascular disease has not yet been clearly elucidated, but is known to have low expression in patients with congestive heart failure and is secreted from migrating cardiac valve interstitial cells. Additionally, it is known that the *miR-206/Fn1* axis inhibits apoptosis in some forms of cancer. Thus, the *Hotair/miR-206/Fn1* axis is similarly thought to prevent apoptosis following AMI [26].

### lncRNAs in heart fibrosis

**Fig. 2:**
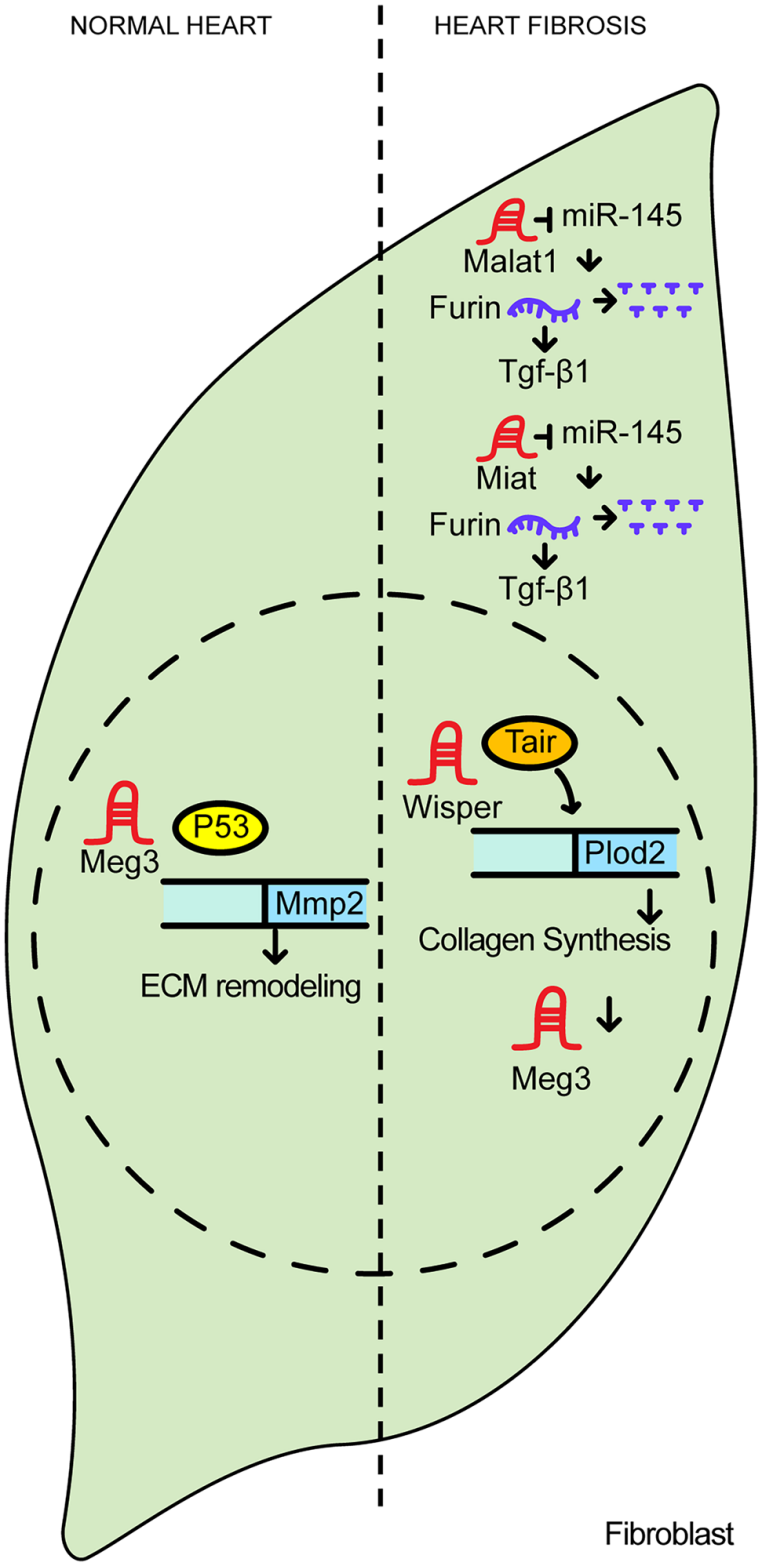
The regulatory functions involved in heart fibrosis

Cardiac fibrosis refers to pathophysiologic event showing increased myofibroblast activity and excessive extracellular matrix accumulation, particularly collagen type I, during cardiac remodeling in most cardiac disease. The principal cause of myocardial interstitial fibrosis is MI but is also observed in hypersensitive heart disease, hypertrophic cardiomyopathy (HCM), and idiopathic dilated cardiomyopathy (DCM) [27]. Cardiac fibrosis can be classified into two main categories: heart failure with reduced ejection fraction (HFrEF) or heart failure with preserved ejection fraction (HFpEF) [28]. In HFrEF, the heart’s ability to generate systolic force is compromised, leading to a decrease in the EF—the proportion of blood expelled with each contraction. On the other hand, in HFpEF, the typical parameters of systolic function are mostly preserved, but diastolic filling and relaxation are impaired [28]. Figure 2 demonstrates the functions of lncRNAs related to heart fibrosis.

In cardiac fibroblasts, some lncRNAs regulate fibrosis-related genes by modulating transcription factor occupancy within gene promoter regions. The lncRNA maternally expressed gene 3 (*Meg3*) is most abundant in the nucleus of cardiac fibroblasts and its expression is downregulated in mice after transverse aortic constriction (TAC). *Meg3* mechanistically decoys p53 by interfering with p53’s ability to bind to the promoter of matrix metallopeptidase 2 (*Mmp2*) and thus allowing the cell to upregulate *Mmp2* expression [29]. As the major role of Mmp2 is extracellular matrix (ECM) remodeling, reduced *Meg3* levels help drive *Mmp2* expression therefore reducing fibrosis and myocardial hypertrophy as well as improving diastolic function in a mouse model of TAC. Therefore, *Meg3* is an attractive target for limiting ECM remodeling during the process of heart fibrosis [30].

Wisp2 super-enhancer-associated RNA (*Wisper*) is a lncRNA that induces fibrosis by acting as guide molecule in the nucleus of fibroblast. *Wisper* is highly expressed in MI animal models as well as in human aortic stenosis patients. When *Wisper* expression is knocked down, cell migration and proliferation decreased, and apoptosis increased when compared to controls. RNA sequencing also revealed that pro-apoptotic genes and cell cycle inhibitors were increased, while pro-inhibitory molecules that promote fibrosis were decreased. Conversely, when *Wisper* was overexpressed, expression of fibrosis-related genes also increased. Specifically, when *Wisper* was delivered intraperitoneally into a mouse model of induced MI, cardiac fibrosis was reduced. The mechanism of how *Wisper* causes fibrosis is that the T-cell intracellular antigen 1 (TIA1)-related/like protein (Tiar) protein binds to *Wisper* and promotes the expression of heart-specific procollagen-lysine,2-oxoglutarate 5-dioxygenase 2 (*Plod2)* gene. Plod2 subsequently induces collagen synthesis and makes the extracellular matrix stable. In sum, the identification of *Wisper* represents crucial step toward anti-fibrotic therapeutic approaches and diagnosis [31].

Sequestering miRNAs is also an important mechanism of lncRNA function in cardiac fibrosis. In the mouse MI model, lncRNA metastasis-associated lung adenocarcinoma transcript 1 (*Malat1*) increases sixfold when compared to controls. Loss of *Malat1* attenuated the cardiac dysfunction induced by MI, resulting in the recovery of the percentages of fractional shortening (FS) and ejection fraction (EF). When MI hearts were transduced with *Malat1* siRNA using lentiviral delivery, infarct size and fibrotic area were both reduced. In addition, the expression levels of collagen I and collagen III, which are fibroblast markers, were decreased. Angiotensin II (AgII) is known to be involved in both fibroblast proliferation and myofibroblast transdifferentiation in the heart. When *Malat1* was decreased in AgII-treated neonatal mouse cardiac fibroblasts (NMCFs), the levels of collagen and alpha-smooth muscle actin were reduced. Conversely, *Malat1* promotes cardiac fibrosis after MI by sponging miR-145. miR-145 functions in the suppression of transforming growth factor-beta1 (TGF-β1) by reducing Furin expression. In sum, *Malat1* appears to cause cardiac fibrosis by limiting miR-145-dependent suppression of TGF-β1 [32].

Abnormal increases in the lncRNA myocardial infarction-associated transcript (*Miat*) are observed in the MI mouse model which results in cardiac interstitial fibrosis. Conversely, ectopic expression of siRNA targeting *Miat* in the MI mouse model restores the normal ratio of EF and FS. In addition, infarct size and fibrosis area were reduced in this model. Also, gene expression of collagen I and collagen III were reduced. An in vitro study that treated AgII in NMCF revealed that *Miat* activates *Furin/Tgf-β1* by inhibiting miR-24 as a ceRNA, and that this resulted in cardiac fibrosis [33].

### lncRNAs in cardiomyopathy

Cardiomyopathy manifests as myocardial or electrical dysfunction within the heart [34]. According to the Global Burden of Disease (GBD) 2020 study, cardiomyopathy and myocarditis caused 0.37 million deaths in 2020 and affected 6.11 million people globally during that year [14]. Eight cases out of approximately 100,000 are diagnosed with cardiomyopathy each year and classically fall into three types of cardiomyopathy: hypertropic cardiomyopathy (HCM), dilated cardiomyopathy (DCM), and restrictive cardiomyopathy (RCM).

**Fig. 3:**
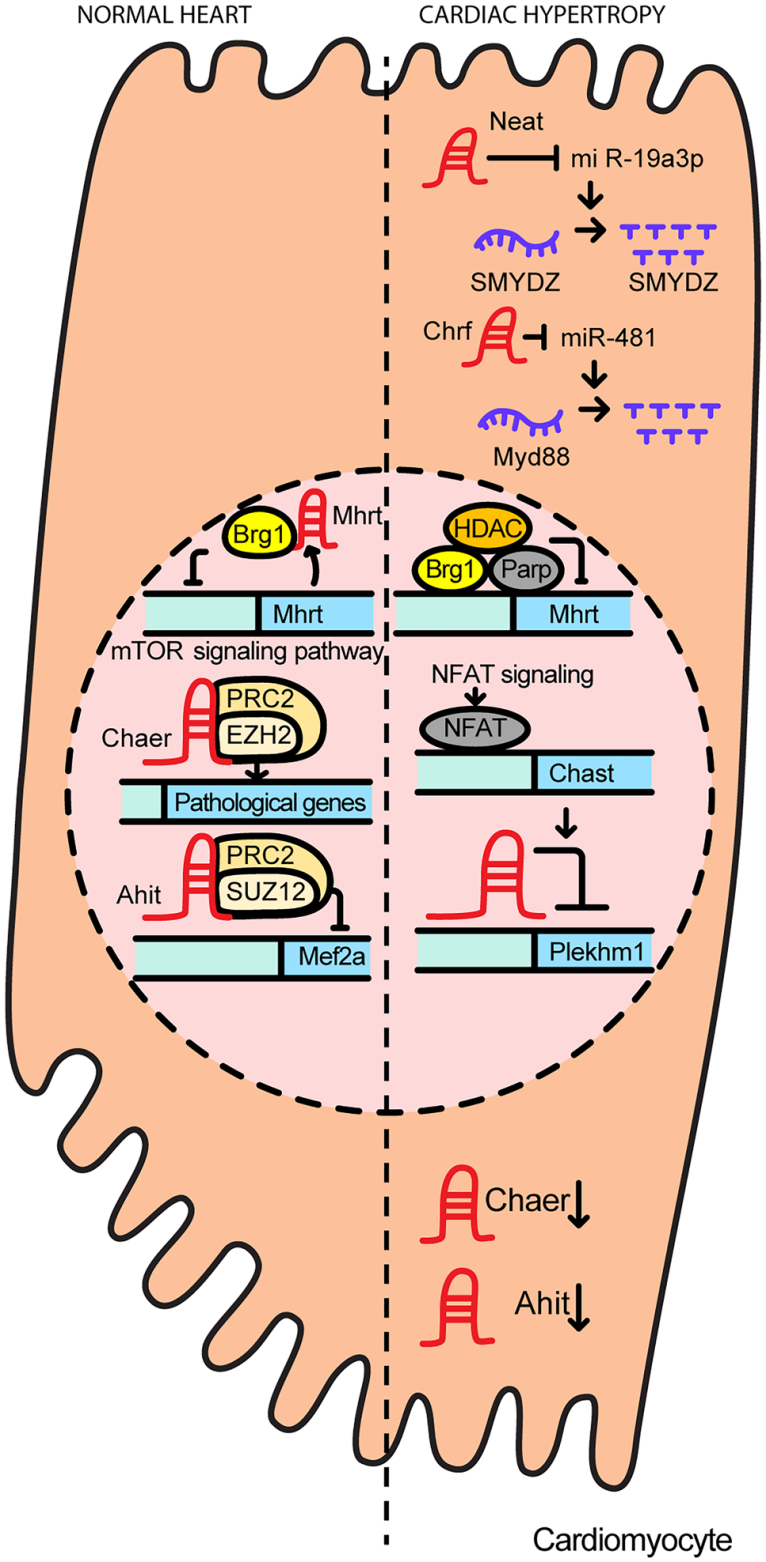
The regulatory functions involved in cardiac hypertrophy

HCM is characterized by marked myocardial hypertrophy without preexisting overload and, in about 50% of cases, it is hereditary in nature [11, 12]. For example, 30–60% of HCM patients have sarcomere variants [14]. HCM can arise at any age, but onset at young age can result in more serious disease symptoms [35]. Figure 3 shows how lncRNAs associated with HCM function.

Some lncRNAs function as scaffolds, guides, and/or signaling molecules within the nucleus during pathogenesis. The myosin heavy chain-associated RNA transcript *myosin heavy-chain-associated RNA transcripts* (*Mhrt*) functions as cardio-protective because it inhibits the development of myocardial hypertrophy. Under pathological stress, BRG1 forms a chromatin repressor complex with histone deacetylase (HDAC) and poly-ADP ribose polymerase (PARP) proteins to suppress *Mhrt* expression. Alternately, *Mhrt* acts as a guide RNA that antagonizes the chromatin remodeling function of brahma-related gene-1 (BRG1) by binding to the BRG1 helicase domain and thereby interfering with genomic targeting of BRG1. Human *Mhrt* has been shown to have a conserved function as a modulator of myocardial function during cardiomyopathy. In hearts under stress, including those with hypertrophic, ischemic, or idiopathic cardiomyopathy, there was a significant decrease in *Mhrt* levels, with reductions of 82.8%, 72.8%, and 65.9% observed in in vitro studies of human heart. Another study showed that patients with chronic heart failure demonstrated significantly lower expression levels of *Mhrt* in plasma when compared to healthy individuals (*p* < 0.05). Additionally, individuals with higher levels of *Mhrt* exhibited a significantly improved overall survival rate when compared to those with lower levels of this lncRNA [36–39].

The lncRNAs *antihypertropic interrelated transcript(Ahit)* and *cardiac-hypertropy associated factor (Chaer)* function as scaffold molecules. *Ahit* is maximally expressed at 6 weeks in hypertrophy-induced TAC-operated mice. When *Ahit* was overexpressed under hypertrophic stress, increases in cell size and hypertrophic markers such as atrial natriuretic peptide (ANP), brain natriuretic peptide (BNP), and beta-myosin heavy chain (β-MHC) were blunted. This anti-hypertrophic effect of *Ahit* is due to direct *Ahit* binding to suppressor of zeste 12 protein homolog (SUZ12), a component of the polycomb repressor complex 2 (PRC2) complex, and this event blocks the regulation of *myocyte enhancer factor 2A (Mef2A)* in *cis*. *Ahit* also has a human homolog, the leukemia-associated noncoding IGF1R activator RNA1 (LUNAR1) [38].

The lncRNA *Chaer* was found to be downregulated in the pressure overload-induced mouse model. When *Chaer* is diminished, hypertrophic growth of cardiomyocytes is inhibited and hypertrophic genes such as natriuretic peptide A (*Nppa*), *β-MHC*, and skeletal muscle alpha-actin (*Acta1*) are reduced. In contrast, overexpression of *Chaer* induces cardiomyocyte enlargement. After hypertrophic stimulation, the mammalian target of rapamycin (mTOR) signaling pathway-dependent lncRNA *Chaer* reduces H3K27 trimethylation of genes linked to pathological changes by binding to enhancer of zeste 2 polycomb repressive complex 2 subunit (EZH2), a methyltransferase subunit of PRC2. Moreover, *Chaer* is conserved in both rodents and humans [40].

The lncRNA cardiac hypertrophy-associated transcript (*Chast*) functions as a signaling molecule. Overexpression of *Chast* results in an increased cardiomyocyte size and expression of hypertrophic markers. *Chast* has a binding site for nuclear factor of activated T cells (NFAT), a pro-hypertrophic transcription factor, within its promoter region, and is upregulated by activated NFAT signaling. By suppressing *pleckstrin homology and RUN domain containing M1 (PlekhM1)*, cardiomyocyte autophagy is prevented, and hypertrophy is induced by *Chast*. *Chast* has a homologous sequence in humans and the human form is also upregulated in patients with aortic stenosis that drives cellular hypertrophy [41].

The majority of lncRNAs associated with cardiac hypertrophy function within the cytosol. The upregulation of *cardiac hypertropy related factor* (*Chrf*) is known to induce apoptosis in cardiomyocytes, and downregulation of *Chrf* decreases ANF and β-MHC levels, both of which are hypertrophic-related genes. *Chrf* also increases the myeloid differentiation primary response gene 88 (Myd88) protein by sponging the miRNA miR-489. In addition, the sequence of *Chrf* is not fully conserved between species, but the miR-489 binding site of *Chrf* is conserved between mouse and human. Further, the expression of human *Chrf* is increased in human heart failure [38]. The lncRNA *nuclear paraspeckle assembly transcript 1* (*Neat1*) also functions as a sponge for miR-19a-13p and thus prevents miR-19a-13p from quenching the [Su(var)3–9, enhancer-of-zeste and trithorax] (SET) and myeloid, nervy, and DEAF-1 (MYND) domain-containing protein SET and MYND domain containing 2 (Smyd2) mRNA transcript. Through this mechanism, *Neat1* upregulates Smyd2 to generate and promote cardiac hypertrophy [42].

The lncRNA *phospholipid scramblase 4* (*Plscr4*) is expressed at a higher levels in cardiomyocytes than in cardiac fibroblasts. It is increased in TAC mice and cardiomyocytes treated with Ang II to create a hypertrophic state. *Plscr4* plays a role in preventing hypertrophy and in induced hypertrophy models, depletion of *Plscr4* results in an increase in the hypertrophic markers ANP, BMP, β-MHC, as well as hypertrophic cardiomyocytes. Overexpression of *Plsr4* attenuates hypertrophic markers and mitigates cardiac hypertrophy in induced cardiac hypertrophy. *Plscr4* acts as an endogenous sponge and sequesters miR-214 which functions in suppressing the protein mitifusion2 (Mfn2). In the presence of *Plscr4*, miR-214 is unable to function resulting in Mfn2 activation. Mfn2 is located in the mitochondrial outer membrane and serves as a negative regulator of cardiac hypertrophy by participating in mitochondrial fusion. Thus, lncRNA *Plscr4* represses cardiac hypertrophy through the miR-214/Mfn2 axis [42]

**Fig. 4:**
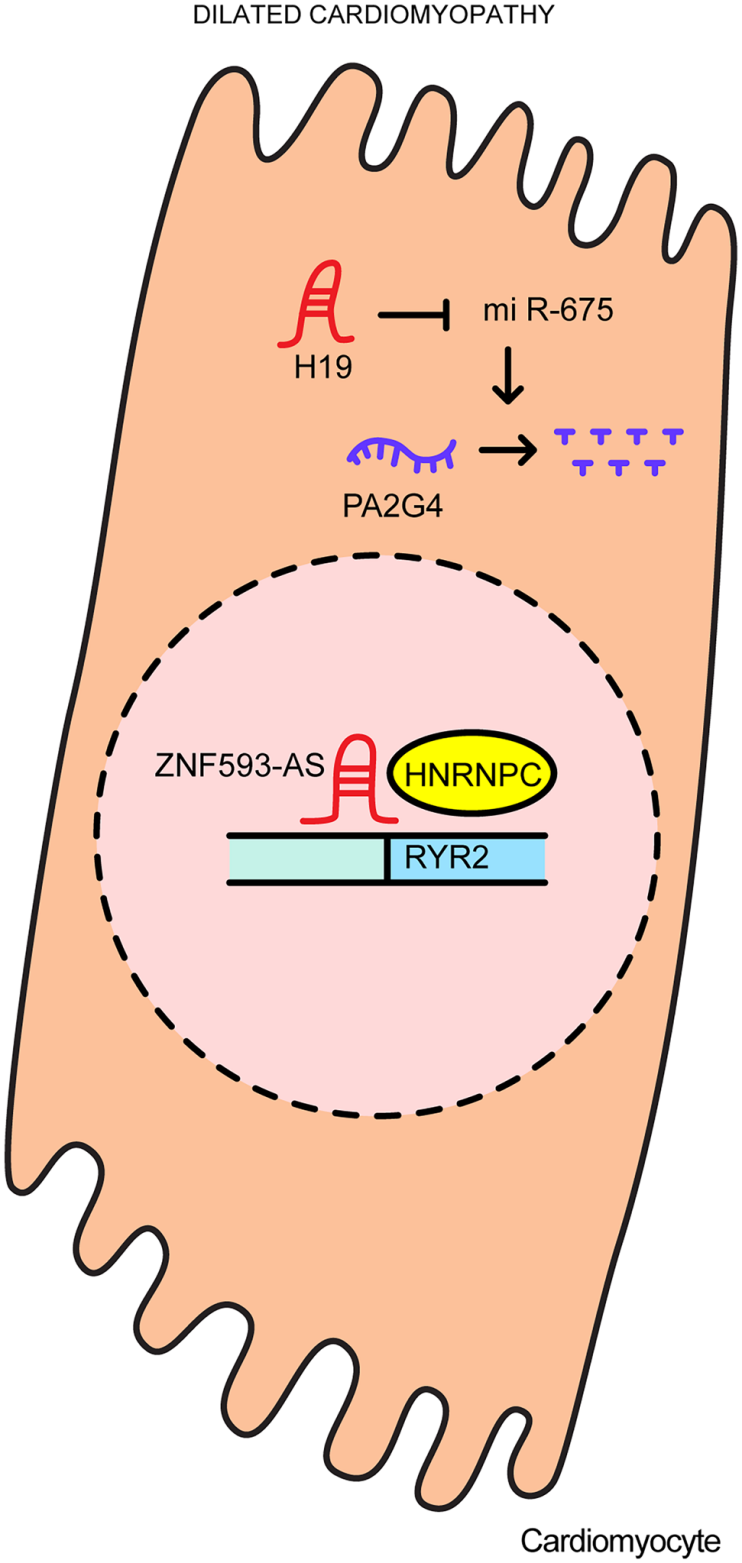
The regulatory functions involved in dilated cardiomyopathy

DCM refers to cardiomyopathy characterized by systolic failure with myocardial thinning and ventricular enlargement, and most often occurs before the age of 50. Abnormal heart rhythms or heart valve abnormalities complicate the course of DCM [43, 44]. Figure 4 shows how lncRNAs associated with DCM function.

The lncRNA *ZNF593-AS* (ENST00000448923.2) has been identified in patients with DCM. Decreased expression of *ZNF593-AS* results in loss of cardiac contractile function because *ZNF593-AS* guides for heterogeneous nuclear ribonucleoproteins C1/C2 (HNRNPC) to stabilize the ryanodine receptor 2 (RYR2) mRNA transcript. RYR2 is a major component of the calcium receptor present in the sarcoplasmic reticulum and is involved in calcium handling and the contractility of cardiac muscle. Moreover, the destabilization of RYR2 by *ZNF593-AS* depletion results in abnormalities in cardiac contractility [45].

The lncRNA *H19* is encoded by a 2.7 kb gene that is maternally expressed and paternally imprinted. It is located close to the telomeric region of chromosome 11p15.5 and is mutually imprinted and regulated with its adjacent gene, insulin-like growth factor 2 (*IGF2*) [42–44]. The expression of lncRNA *H19* is increased in a mouse model of DCM,and when *H19* is lost, the rate of apoptosis in cardiomyocytes is reduced and cardiac function is improved. During DCM, *H19* sequesters miR-675 which, in turn, inactivates proliferation-associated 2G4 (PA2G4), a potential regulator of receptor tyrosine-protein kinase erbB-2 (ErbB3) signaling that promotes cell differentiation, apoptosis, and cell growth. In sum, the *H19/*miR-675/PA2G4 axis appears to significantly regulate doxorubicin-induced DCM [46].

## lncRNAs in cardiac proliferation and development

So far in this manuscript we have discussed how lncRNAs function in CVD and which may potentially be therapeutic targets. We now direct our attention to lncRNAs that possess restorative capabilities from a slightly different perspective. Studies using nuclear bomb tests with carbon-14 have been performed on genomic DNA harvested from human myocardial cells. Cardiomyocytes increase by 1% per year at the age of 25, and this decreases to 0.45% by age 75. Thus, the limited ability of cardiomyocytes to regenerate markedly declines with age [47]. Cardiomyocyte loss and insufficient cardiomyocyte regeneration result in most CVDs, especially in MI. Infarction-induced heart failure results in a 25% reduction in left ventricle cardiomyocytes and leads to the death of approximately up to 1 billion myocardial cells [48]. Therefore, we investigated how lncRNAs can promote cardiomyocyte proliferation or promote pre-existing cardiomyocytes (i.e., endogenous regeneration) as a way to regenerate heart tissue. We also examined the lncRNAs involved in cardiac differentiation that enable exogenous transplantation [49].

**Fig. 5:**
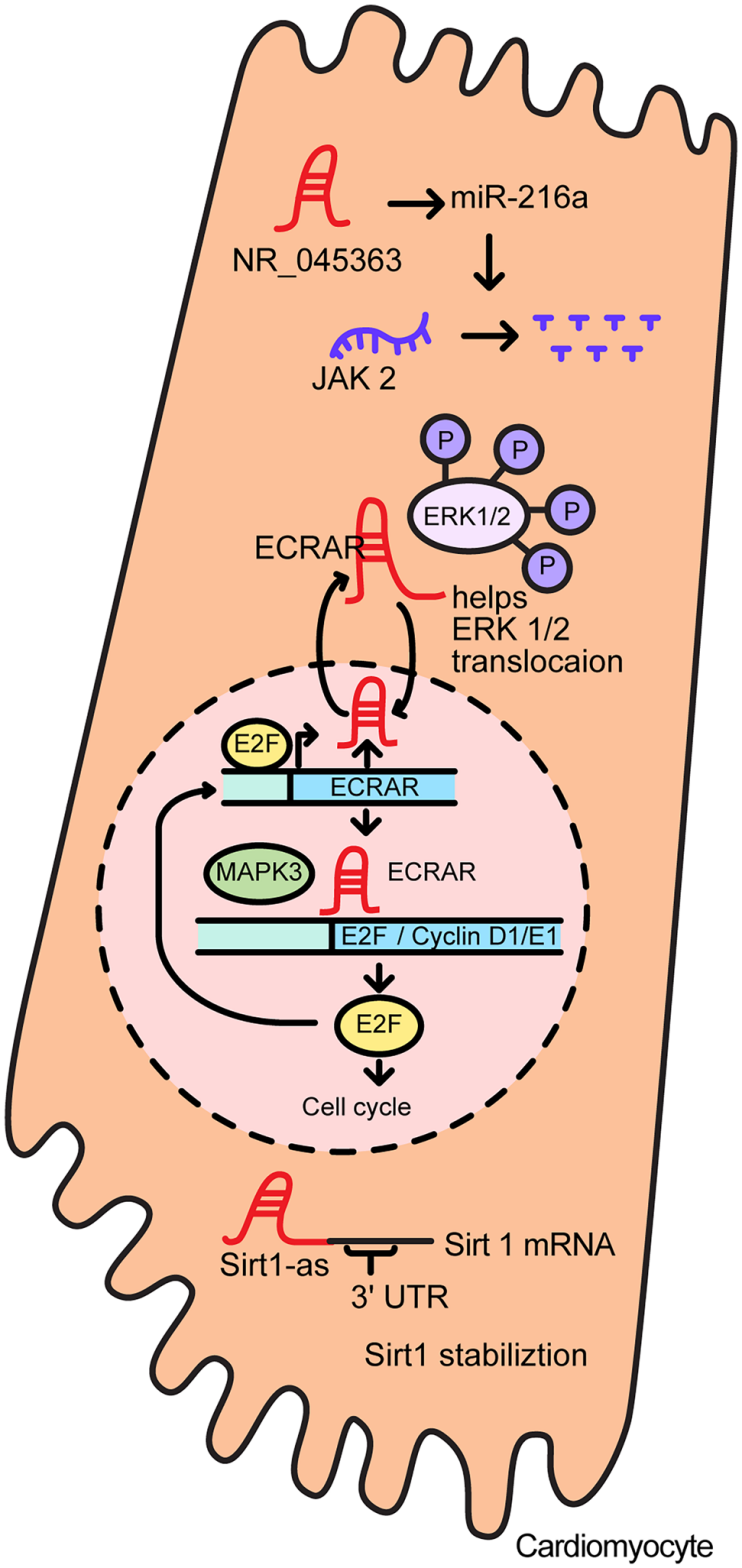
The regulatory functions involved in activating cardiomyocyte proliferation

Several lncRNAs activate, or repress, cardiomyocyte proliferation both within and outside of the nucleus. Figure 5 illustrates the mechanism by which lncRNAs activate cardiomyocyte proliferation. Examples of lncRNAs that activate cardiomyocyte proliferation in the nucleus include *ECRAR*, *NR_045363*, and *Sirt1-as*. The lncRNA endogenous cardiac-associated regulator (*ECRAR*), a signal-regulating lncRNA, is significantly upregulated in fetal heart when compared to adult heart. Ectopic expression of *ECRAR* in rat ventricular cardiomyocytes promoted cardiomyocyte proliferation. Also, the mitosis-specific histone mark, phosphorylated histone H3 (pH3), was increased. Although *ECRAR* promotes cardiac proliferation, it does not appear to induce cardiac hypertrophy. Cardiomyocyte proliferation provoked by *ECRAR* occurs through the extracellular signal-regulated protein kinases 1 and 2 (ERK 1/2) pathway, which plays an important role in cell cycle regulation. Positive feedback occurs when E2F transcription factor 1(E2F1), a downstream protein target of *ECRAR*, elevates *ECRAR* expression by binding within the *ECRAR* promoter [50].

Other lncRNAs are involved in mRNA stabilization and miRNA binding within the cytoplasm. The lncRNA Sirt1 antisense (*Sirt1-as*) lncRNA, which is highly expressed in embryonic mouse hearts, overlaps with a region within the Sirt1 mRNA 3′ UTR. Overexpression of *Sirt1-as* has been shown to promote cardiomyocyte proliferation and increase the proliferation marker Ki67, with identical results obtained both in vivo and in vitro. When *Sirt1-as* was injected into an MI mouse model, it increased the animal survival rate from 56.7 to 83.3%, FS and EF, while reducing infarct size compared to controls. *Sirt1-as* also inhibits apoptosis and decreases cardiomyocyte size due to *Sirt1-as* binding, and consequential stabilization of the Sirt1transcript. Adequate levels of Sirt1 in the heart protect this organ from oxidative stress as well as attenuating apoptosis [51].

The highly conserved lncRNA *NR_045363* (1700024F13Rik) is a mouse orthologue of *ENST00000435695* (LOC101927497), an lncRNA that is antisense to human cyclin-dependent kinase 6 (CDK6) mRNA transcript. The expression of this lncRNA is higher in the embryonic mouse heart than in the adult heart and is specifically expressed in cardiomyocytes. In the MI model, mice expressing ectopic *NR_045363* showed an increase in heart regenerative capacity compared to the control group, displayed improved EF and FS, and showed a significantly reduced infarct size. *NR_045363* appears to positively control cardiomyocyte mitotic activity and proliferation by binding miR-216a and activating JAK2-STAT3 phosphorylation [52].

Representative lncRNAs that inhibit cardiomyocyte proliferation include dachshund homolog 1 (*DACH1*) and cardiomyocyte regeneration-related lncRNA (*CRRL*). The lncRNA *DACH1* is a signaling molecule that is highly conserved between mouse and human, and is strikingly upregulated during postnatal heart development. lnc*DACH1* is increased in the hearts of MI mice compared to controls and overexpression of lnc*DACH1* in Myh6-lncDACH1transgenic mouse shows a decreased rate of cardiomyocyte proliferation and reduced cardiac regeneration capacity. Conversely, MI-induced mouse models in mice with cardiac-specific lnc*DACH1* knockout show an increased rate of cardiomyocyte proliferation, improved cardiac function, such as a higher ratio of EF and FS, and reduced infarct size compared to controls. lnc*DACH1* binds to inorganic pyrophosphatase 1 (PPA1) and restricts its dephosphorylation activity and also regulates yes1 associated transcriptional regulator (YAP1) signaling by increasing YAP1 phosphorylation and decreasing its nuclear translocation by binding PP1A [53].

**Fig. 6:**
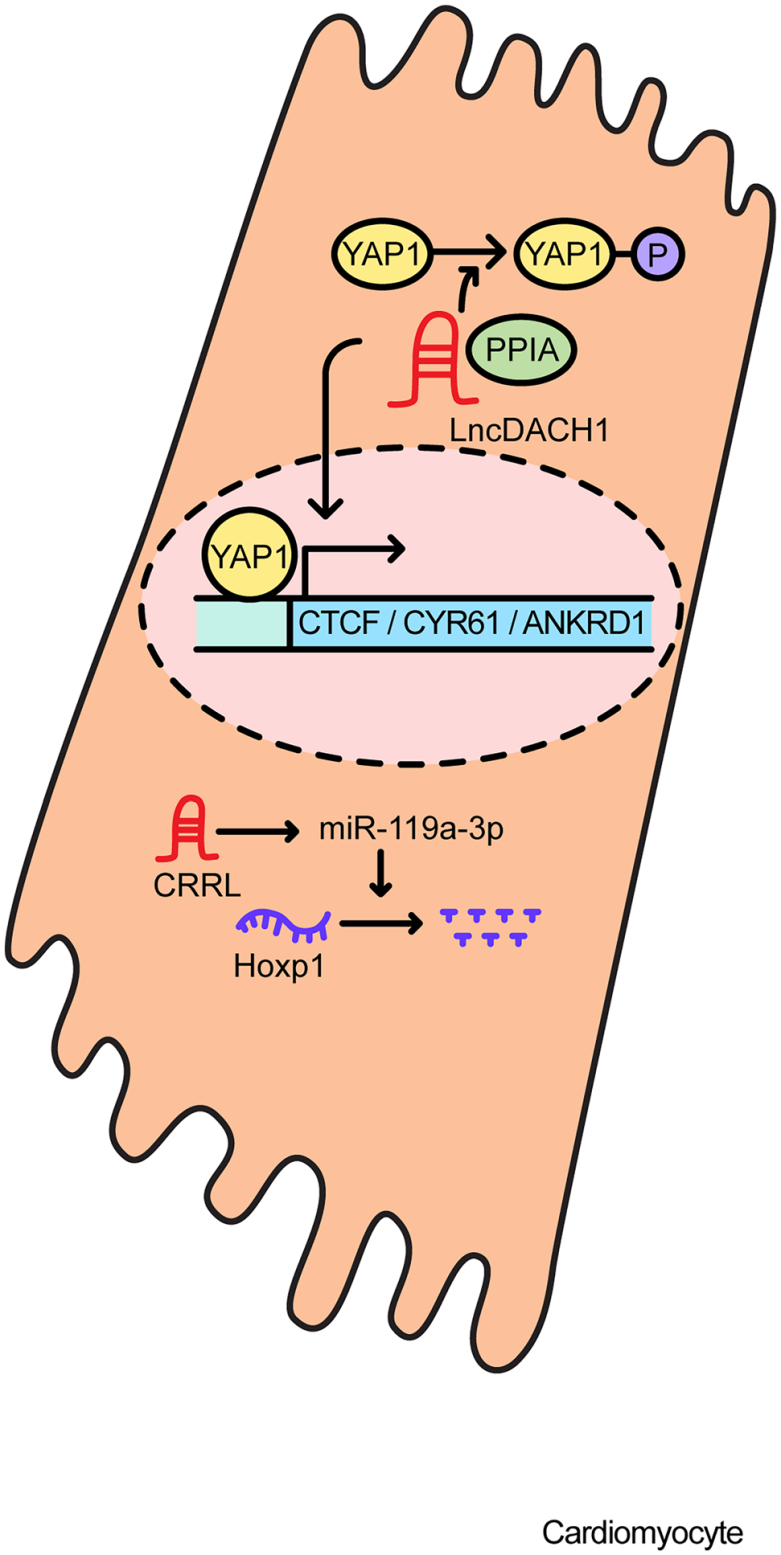
The regulatory functions involved in suppressing cardiomyocyte proliferation

lncRNA *CRRL* (ENST00000525927.5) is markedly upregulated in adult heart cardiomyocytes. In an MI model created by left anterior descending artery (LAD) ligation, loss of *CRRL* using RNA interference restored the ratio of left ventricular EF and left ventricular FS, and reduced infarct size. Also, depletion of *CRRL* in cardiomyocytes resulted in cardiomyocyte proliferation without inducing cardiac hypertrophy. In this setting, *CRRL* appears to act as a ceRNA by sequestering miR-119a-3p. This results in increases in the activity of a target, Hoxp1, which is known to be a key suppressor of embryonic cardiomyocyte differentiation [54]. Figure 6 depicts the mechanisms of lncRNAs that inhibit cardiomyocyte proliferation.

**Fig. 7:**
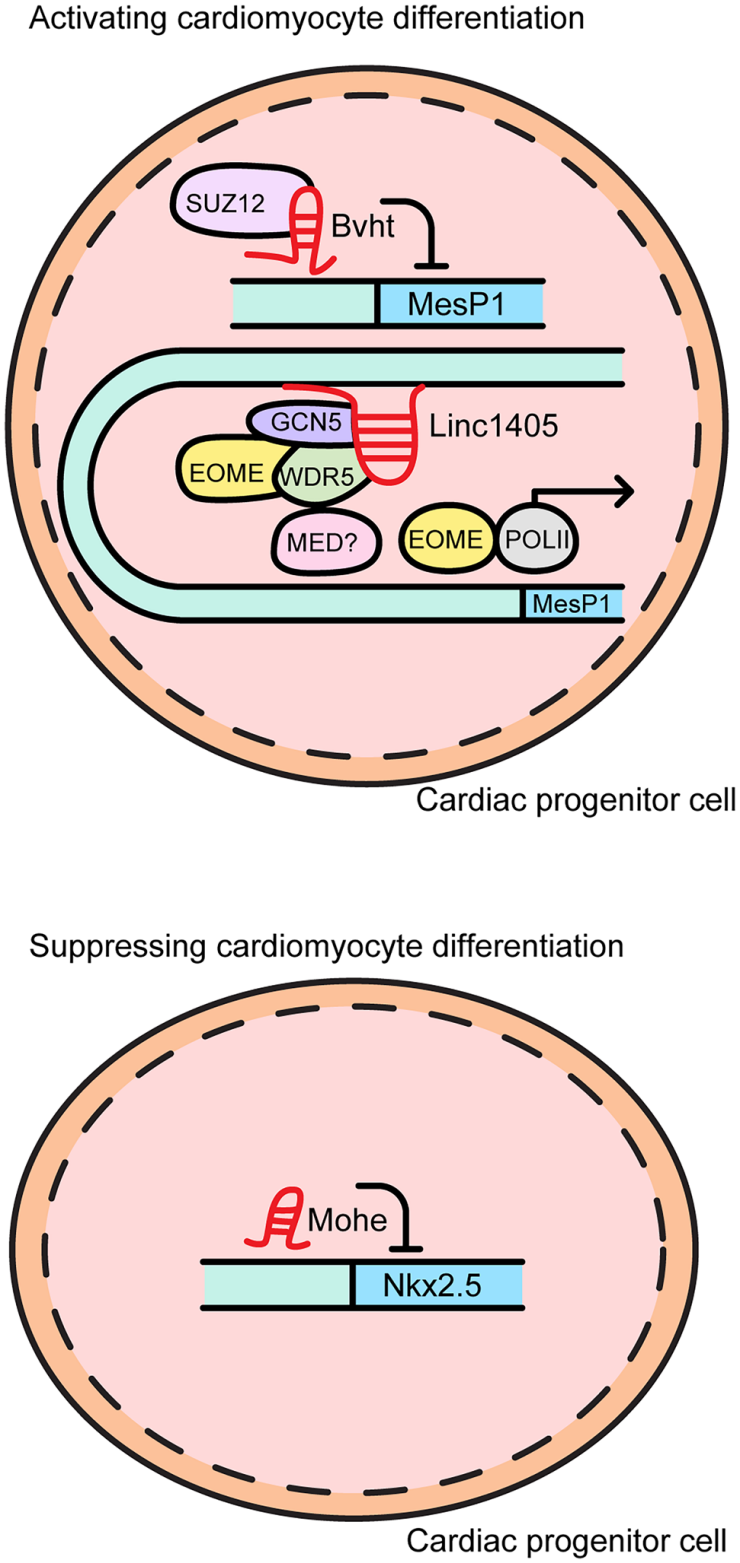
The regulatory functions involved in cardiomyocyte differentiation

Various lncRNAs are required for cardiac differentiation. The manner in which lncRNAs related to cardiomyocyte differentiation function is illustrated in Fig. 7. The lncRNA *Bravehear*t (*Bvht*; AK143250) is located on mouse chromosome 18:61,799,307–61,807,126 (+ strand, mm9) and consists of a ~ 590 nucleotide transcript encoding three exons. It is highly expressed in mouse heart tissue and differentiation of mouse embryonic stem cells (mESCs) to cardiomyocytes shows that *Bvht* regulates a core network in cardiovascular development. *Bvht* plays an important role in the differentiation of nascent mesoderm to cardiomyocytes by inhibiting mesoderm posterior bHLH transcription factor 1 (*MesP1*) through interaction with SUZ12, leading to cardiac lineage commitment [55].

The lncRNA *Linc1405* is transcribed in close proximity to eomesodermin (eomes)*,* a mesoderm gene, and is abundant in heart tissue of the embryo. During cardiac differentiation from mESCs, *Linc1405* acts as a scaffold that drives cardiac differentiation by regulating the *MesP1* gene. Exon 2 of *Linc1405* binds to eomes and the *Linc1405*/eomes complex further complexes with WD repeat domain 5 (WDR5) and general control non-depressible 5 (GCN5) within the enhancer region of *MesP1*. In a mouse model, loss of *Linc1405* resulted in reduced EF and FS, as well as decreased ventricular wall thickness [56].

The lncRNA modulating second heart field progenitor *Moshe*, 1010001N08Rik-203, is located in an antisense orientation to the *GATA binding protein 6* (*Gata6*) gene. It is expressed in the embryonic heart at E8.5, E9.5, and E12.5, and during cardiomyocyte differentiation, *Moshe* suppresses cardiomyocyte differentiation by downregulating secondary heart field genes. *Moshe* further inactivates cardiomyocytes by binding to the *NK2 homeobox 5* (*Nkx2.5*) promoter and *Moshe* is highly conserved among species [57].

## Therapeutic approaches in lncRNA

lncRNAs represent auspicious therapeutic targets in CVD. Their tissue specificity and low expression levels mean that they can be treated with a limited amount of drugs with a resultant low toxicity. Using lncRNA for therapeutic purposes can be done by either removing disease-causing lncRNA or delivering disease-relieving lncRNA to the lesion site.

Therapeutic technologies using lncRNA have been developed mainly in an approach that targets their sequences [58]. siRNAs are short, double-stranded RNAs with 21 base pairs that precisely match their target lncRNA. siRNA are recognized by the RNA-induced silencing complex (RISC) and this results in target RNA degradation. The antisense strand also binds to the complementary target lncRNA leading to argonaut, the core of the RISC, performing endonucleolytic cleavage by P-element induced Wimpy testis (PIWI) domain [59].

As opposed to siRNAs, short hairpin RNA (shRNA) are synthesized in the nucleus of cells, further processed and transported into the cytoplasm, and then associate with RISC for their activity. shRNA can be expressed either transiently or stably [58, 60].

Antisense oligonucleotides (ASOs) are single-stranded RNA molecules of approximately 4 to 10 kDa in mass. ASO contain 13–25 nucleotides in their structure and also contain at least a 6-mer DNA molecule within their central domain. ASOs form ASO/RNA heteroduplexes with lncRNA via their DNA moiety and this leads to endogenous RNAse H-mediated RNA cleavage. Because ASOs do not bind to other factors such as RISC, they act not only on mature RNA transcripts but also on pre-RNA. Moreover, ASO make various chemical modifications possible that result in increased stability, binding force, and cell permeability. For example, LNA (locked nucleic acid), a nucleic acid derivative containing bicyclic furanose unit which bridges oxygen atoms on the 2′ position sugar and 4′ carbon of the ribose increases binding affinity. Other modifications include neutral backbones such as phosphorodiamidate morpholino oligomer, peptide nucleic acid, and phosphorothioates [58, 61–63]. These approaches take advantage of strong target specificity and provide a potentially convenient transition from the laboratory to the clinic. In addition, given current progress in interfering RNA techniques, necessary development time for therapeutic development may prove to be shortened [64].

Other approaches for targeting lncRNAs include small molecules, ribozymes, and deoxyribozymes. Conventional small molecules bind to either highly structured lncRNA or RNA-binding proteins. They alter their secondary or tertiary structure, or mask protein binding sequences within the targeted lncRNA. Therefore, in order to make “druggable” small molecules to target lncRNA, a detailed understanding of the lncRNA and binding protein structure is required [63]. Ribozymes or deoxyribozymes, such as hammerhead ribozyme, are enzymatic nucleic acid molecules that bind to complementary target sequences and catalyze RNA cleavage. These molecules cleave target lncRNAs in a site-specific manner without enzymatic proteins such as RISC or RNase H [58, 65, 66].

lncRNA delivery systems have been developed to deliver therapeutic lncRNAs or lncRNA-targeting molecules into lesions. Lentiviral vectors are the most commonly used lncRNA-carrying delivery systems as they have a packing capacity of up to 8 kb [67] and can infect both dividing and non-dividing cells. Lentiviral vectors also have a higher transduction rate and lead to non-viral protein expression after transduction. Due to integration into the genome, lentiviral vectors may provide stable long-term transgene expression [68].

Adenoviral vectors are also widely used for lncRNA overexpression and suppression. These viruses have a packing capacity of up to 5 kb and can transduce both dividing and non-dividing cells similar to lentiviral vectors. Adenovirus is a non-encapsulated, linear double-strand DNA virus [69], and therefore, no genomic integration occurs and the expression is transient in nature. Even though lentiviral vectors are not used in clinical trials, adenovirus vectors have found use in the clinic [70].

Non-viral vectors, such as liposomes, are the earliest version of lipid nanoparticle (LNP) delivery platforms. Compared with viral vectors, liposomes reduce immunogenicity and toxicity by not integrating into the genome, and can provide short-term, transient, and high-level expression of transported materials, including nucleic acids, small molecules, and proteins. Liposomes have versatile features for transporting hydrophobic and hydrophilic molecules via endocytosis, and this allows them to transduce both dividing and non-dividing cells [69, 70]. Numerous liposomal drugs have been successfully applied in clinical practice [71].

The next generation of nanoparticles (NP) consists of nucleic acid complexes with cationic lipid, nanostructured lipid carriers, peptides, polymers, and polysaccharides. These delivery systems remain solid at physiological temperatures and exhibit enhanced stability [69, 70]. Because LNPs can control the accurate location and duration of the delivered drug, they are suitable for various drug delivery applications [71].

Recently, endogenous delivery systems have been actively studied. Exosomes are extracellular vesicles (EVs) ranging in diameter from 30 to 200 nm (average ~ 100 nm), which can fuses with the cell’s plasma membrane [69]. Johnstone et al. discovered EVs in adult sheep reticulocytes in 1983. These structures were initially termed exosomes [72] and were thought to be cellular debris. Since these seminal studies, accumulating evidence shows that exosomes contain DNA, mRNA, miRNA, and lncRNA, and facilitate communication between cells [73]. They directly engage with a receptor on target cells, can transfer the receptor of the origin cell to the recipient cell, and deliver the contents of the vesicle. By this mechanism, EVs regulate cell-to-cell communication [74] and these characteristics suggest that EVs can be used for lncRNA delivery. Compared to viral or non-viral exogenous vesicles, exosomes can package drugs using patient-derived cells as the source, resulting in lower immunogenicity and higher stability. Exosomes can also be combined with liposomes, organic nanoparticles, or organic nanoparticles, such as exosome-liposome hybrids, to increase target specificity and delivery system controllability [75, 76].

## Conclusions

Despite technical advances in cardiovascular medicine and well-designed emergency medical systems, CVD and especially AMI remain the leading cause of mortality and morbidity in western countries [77, 78]. Immediate reperfusion therapy can save many lives, but reperfusion often induces reperfusion injury which is thought to be mediated by increased oxygen free radicals [79–82]. Complex molecular pathway makes the issue of selecting the most appropriate therapeutic target more complicated, resulting in a lack of effective treatments to reduce reperfusion injury at the present time [83–85]. Similarly, many trials were attempted to regenerate cardiomyocyte by infusion or direct injection of pluripotent stem cells. Carbone et al. summarized 95 studies related to acute myocardial infarction from 2000 to 2020, categorizing those that showed benefits, as well as those that showed no effect or uncertain benefits [86–89]. Due to safety and efficacy problems, there are no stem cell therapies specifically approved by the US Food and Drug Administration (FDA) for the treatment of heart disease, even though a large number of clinics offer various cellular treatments without having gone through the FDA approval process [90]. Therefore, clinical benefit is ambiguous. It is time to renew interest in novel therapeutic targets and develop more novel approaches for better treatment of CVD. In this article, we have highlighted molecular bases of CVD in detail and suggested potential value of lncRNA as well selected molecules as therapeutic targets.

Once characterized as biological noise, lncRNA are now recognized as an important therapeutic target. Growing evidence indicates the principal roles of lncRNAs in complicated regulatory networks that govern cardiac dysfunction and regeneration. Many lncRNAs can be used as crucial therapeutic targets for CVDs, including cardiac hypertrophy, DCM, MI, and cardiac fibrosis. The concept that cardiomyocyte proliferation might be enhanced to improve cardiac regeneration led us to catalog and discuss many lncRNAs that are important in orchestrating this process. Advances in our understanding of lncRNA mechanisms involving cardiac dysfunction and cardiac regeneration underpin the potential for lncRNA therapies.

Natural molecules that target lncRNAs in specific diseases are known. For example, resveratrol targets *metastasis associated lung adenocarcinoma transcript 1* (*Malat1)* in Parkinson’s disease [91] and *noncoding nuclear-enriched abundant transcript 2 (Neat1)* in myeloma [92]. Curcumin targets *H19* in gastric cancer cells [93] and *Malat1* in colon cancer cells [94]. Additionally, berberine targets 538 lncRNAs in non-alcoholic fatty liver disease [95]. More natural compounds targeting lncRNAs can be found on the Clinical Trial.gov website; however, it is important to note that natural compounds do not usually target lncRNAs as their principal target.

The good news is that RNA therapy is advancing. In the past few decades, significant efforts have been made to introduce RNA-based therapeutics into the clinic. As a result, several RNA-based therapeutics such as Eteplirsen, Nusinersen, and Inotersen, each of which is based on ASO technology, have been approved by the Food and Drug Administration (FDA) [96, 97]. This underscores the clinical potential of lncRNA targeted therapy.

However, in order for RNA-based therapy to be successfully applied clinically and commercialized, several limitations must be recognized and improved before drugs targeting lncRNAs can be used to treat CVDs. Even if lncRNAs expressed in human diseases are discovered, animal experiments are required before going to clinical trial, but lncRNAs are generally poorly conserved among species. In addition, even if lncRNAs are conserved in humans and mice, responses may differ between species. Another limitation is the issue of immunogenicity. As a viral defense mechanism, immune response recognizes foreign RNAs via various pathogen-associated molecular pattern (PAMP) receptors. Further, nucleic acid-based therapy can cause off-target effects. For example, sugar moiety modification can increase the probability of off-target cleavage. In fact, only 10–50% of the designed ASOs decrease the intended target [98]. The final hurdle is drug delivery. Therapeutic RNA must cross the cell membrane and, even if it passes through the cell membrane, the lncRNA must escape the endosome to enter the cytoplasm. LNPs, polymers, RNA conjugation, NPs, and virus-based approaches are under development to improve the efficacy of delivery problems. For lysosome rupture, neutrally charged ionizable lipid can be used to release nucleic acid-based drugs more easily [58, 64, 99, 100].

In spite of these difficulties, ‘third-generation gene therapy,’ which aims to treat diseases by artificially regulating gene expression at the transcription level, as exemplified by drugs like Eteplisen (Exondys 51^TM^), Nusinersen, and Inostersen mentioned above [96, 97], will overcome the limitations of many protein-targeted drugs. Currently, drugs targeting only mRNA and miRNA have been released, but in the near future, drugs targeting lncRNAs, especially those important in CVD pathogenesis, are expected to emerge.

## Abbreviation

ENCODE: The Encyclopedia of DNA Elements
lncRNAs: Long noncoding RNAs
ceRNA: Competing endogenous RNA
TWAS: Transcriptional genome-wide studies
CVD: Cardiovascular disease
WHO: World Health Organization
NCDs: Non-communicable diseases
NHANES: National Health and Nutrition Examination Survey
GBD: Global Burden Disease
MI: Myocardial infarction
ARIC: Atherosclerosis Risk in Communities
Snhg1: Small nucleolar RNA host gene 1
PTEN: Phosphatase and tensin homolog
PI3K: Phosphatidylinositol 3′-kinase
VEGFA: Vascular endothelial growth factor A
Phb2: Prohinin2
LDH: Lactate dehydrogenase
Oip5-as1: Opa-interacting protein 5-antisense transcript 1
Sirt1: Sirtuin-1
Ampk: AMP-activated protein kinase
Pgc1-α: Peroxisome proliferator-activated receptor gamma coactivator 1-alpha
HFrEF: Heart failure with reduced ejection fraction
HFpEF: Heart failure with preserved ejection fraction
Meg3: Maternally expressed gene 3
Mmp2: Matrix metallopeptidase 2
TAC: Transverse aortic constriction
ECM: Extracellular matrix
Wisper: Wisp2 super-enhancer-associated RNA
TIA1: T-cell intracellular antigen 1
Tiar: T-cell intracellular antigen 1 (TIA1)-related/like protein
Plod2: Procollagen-lysine,2-oxoglutarate 5-dioxygenase 2
Malat1: Metastasis-associated lung adenocarcinoma transcript 1
FS: Fractional shortening
EF: Ejection fraction
AgII: Angiotensin II
NMCFs: Neonatal mouse cardiac fibroblasts
TGF-β1: Transforming growth factor-beta1
Miat: Myocardial infarction-associated transcript
GBD: Global Burden of Disease
HCM: Hypertropic cardiomyopathy
DCM: Dilated cardiomyopathy
RCM: Restrictive cardiomyopathy
HDAC: Histone deacetylase
PARP: Poly-ADP ribose polymerase
Mhrt: Myosin heavy-chain-associated RNA transcripts
BRG1: Brahma-related gene-1
Ahit: Antihypertropic interrelated transcript
Chaer: Cardiac-hypertropy associated factor
ANP: Atrial natriuretic peptide
BNP: Brain natriuretic peptide
β-MHC: Beta-myosin heavy chain
SUZ12: Suppressor of zeste 12 protein homolog
Mef2A: Myocyte enhancer factor 2A
LUNAR1: The leukemia-associated noncoding IGF1R activator RNA1
Nppa: Natriuretic peptide A
Acta1: Skeletal muscle alpha-actin
mTOR: Mammalian target of rapamycin
EZH2: Enhancer of zeste 2 polycomb repressive complex 2 subunit
PlekhM1: Pleckstrin homology and RUN domain containing M1
Chrf: Cardiac hypertropy related factor
Neat1: Nuclear paraspeckle assembly transcript 1
MYND: Myeloid, Nervy, and DEAF-1
Smyd2: SET and MYND domain containing 2
Plscr4: Phospholipid scramblase 4
RYR2: Ryanodine receptor 2
PA2G4: Proliferation-associated 2G4
ErbB3: Receptor tyrosine-protein kinase erbB-2
ECRAR: Endogenous cardiac-associated regulator
pH3: Phosphorylated histone H3
ERK1/2: Extracellular signal-regulated protein kinases 1 and 2
E2F1: E2F transcription factor 1
Sirt1-as: Sirt1 antisense
CDK6: Cyclin-dependent kinase 6
DACH1: Dachshund homolog 1
PPA1: Pyrophosphatase 1
YAP1: Yes1 associated transcriptional regulator
MesP1: Mesoderm posterior bHLH transcription factor 1
Eomes: Eomesodermin
WDR5: WD repeat domain 5
GCN5: General control non-depressible 5
Nkx2.5: NK2 homeobox 5
RISC: RNA-induced silencing complex
PIWI: P-element induced Wimpy testis
shRNA: Short hairpin RNA
ASOs: Antisense oligonucleotides
LNA: Locked nucleic acid
LNP: Lipid nanoparticle
NP: Nanoparticles
EVs: Extracellular vesicles
MALAT 1: Metastasis associated lung adenocarcinoma transcript 1
NEAT2: Noncoding nuclear-enriched abundant transcript 2
FDA: Food and Drug Administration
PAMPs: Pathogen-associated molecular patterns
